# Impact of maxillomandibular atresia on the response to mandibular advancement devices in obstructive sleep apnea: a scoping review

**DOI:** 10.1007/s11325-026-03783-z

**Published:** 2026-09-25

**Authors:** Izabella P. Manetta, Bruno B. Duarte, Michel B. Cahali

**Affiliations:** 1 Department of Otolaryngology. Hospital das Clinicas HCFMUSP, Faculdade de Medicina, Universidade de Sao Paulo, Sao Paulo, Brazil; 2 Department of Otolaryngology Head and Neck Surgery, Pontifical Catholic University of Campinas, Campinas, São Paulo Brazil

**Keywords:** Obstructive sleep apnea, Mandibular advancement device, Treatment outcomes, Transverse craniofacial deformity

## Abstract

**Purpose:**

To map the existing evidence on the impact of transverse craniofacial alterations on mandibular advancement device treatment outcomes in adults with obstructive sleep apnea, with the objective of identifying knowledge gaps and informing future research and clinical decision-making.

**Methods:**

A systematic literature search was conducted in six databases. Additionally, gray literature sources were searched, and clinical trial registries, including ClinicalTrials.gov, were screened for RCT protocols. The reference lists of all included sources of evidence were also screened, and experts in the field were consulted.

**Results:**

The literature search yielded a total of 1,508 references after duplicate removal. Nine studies and one conference report were found eligible for inclusion. The literature demonstrated heterogeneous associations between transverse craniofacial characteristics and mandibular advancement device treatment outcomes. Most studies (*n* = 7) did not identify significant associations between isolated transverse craniofacial measurements and treatment response. Greater mandibular transverse dimensions were associated with better therapeutic outcomes in two studies, whereas one study reported better treatment response in individuals with smaller mandibular transverse dimensions. Reduced maxillary width and unfavorable anatomical balance were more frequently associated with greater obstructive sleep apnea severity or poorer treatment outcomes.

**Conclusions:**

While some studies suggest that the response to mandibular advancement devices in obstructive sleep apnea is associated with transverse mandibular dimensions, most investigations have not identified this relationship. Future standardized and well-designed studies are needed to clarify the influence of this anatomical characteristic on the outcomes of this common treatment for obstructive sleep apnea.

**Supplementary Information:**

The online version contains supplementary material available at https://doi.org/10.1007/s11325-026-03783-z.

## Introduction

Obstructive sleep apnea (OSA) is a common and chronic disorder characterized by recurrent upper airway collapse during sleep that leads to intermittent hypoxia, sleep fragmentation, and intrathoracic pressure changes [1]. It is estimated to affect approximately 936 million adults aged 30–69 years worldwide, including nearly 425 million individuals with moderate-to-severe disease, highlighting its substantial global health burden [1]. The most common nocturnal symptoms include snoring and observed breathing pauses, while daytime symptoms are excessive sleepiness, fatigue, cognitive deficits, and reduced quality of life. The severity of OSA is classified by the apnea-hypopnea index (AHI), representing the number of apnea (total collapse) and hypopnea (partial collapse) events per hour of sleep. AHI values between 5 and 15 indicate mild OSA, 15–30 moderate, and above 30 severe. The gold standard diagnosis is full-night laboratory polysomnography, which assesses the frequency of respiratory events, desaturation, sleep fragmentation, among other features [2]. OSA is associated with several negative health outcomes such as increased risk of hypertension, cardiovascular diseases, arrhythmias, stroke [3, 4], cognitive and metabolic changes, as well as significant social and neuropsychological impact [5]. Beyond conventional measures of OSA severity, clinically relevant outcomes such as blood pressure, daytime sleepiness, and sleep quality further illustrate the multidimensional burden of the disorder [6] The standard OSA treatment is continuous positive airway pressure (CPAP). CPAP is effective in reversing the upper airway obstruction and hypoxia which subsides this disorder. However, while CPAP efficacy in reversing OSA symptoms has been demonstrated [7], adherence is often sub-optimal with several interferences [8].

Mandibular advancement device (MAD) therapy is widely used in the management of adults with mild to moderate obstructive OSA [9]. In patients with severe OSA, MADs may constitute an alternative therapeutic option in cases of CPAP intolerance [9].

However, treatment response to MAD is variable and reflects the complex pathophysiology of OSA, which is influenced by individual anatomical and non-anatomical traits as well as overall health status [10]. These factors, often conceptualized as OSA phenotypic traits, emphasize the heterogeneity of the disorder and reinforce the need for individualized therapeutic approaches [11, 12].

Transverse craniofacial discrepancies are clinically relevant to obstructive sleep apnea because the dimensions of the maxillary and mandibular skeletal framework may influence the space available for the tongue, the patency of the nasal cavity, and the mechanical stability of the pharyngeal airway [13]. Maxillary constriction and maxillomandibular transverse deficiency may reduce oral cavity volume, promote posterior tongue displacement, and decrease retrolingual airway space, thereby increasing upper airway collapsibility during sleep. Transverse maxillary deficiency may also be associated with increased nasal resistance and a greater tendency toward oral breathing, which can further compromise upper airway stability. Recent evidence has shown that reduced transverse maxillary dimensions are associated with greater pharyngeal collapsibility during drug-induced sleep endoscopy, supporting the potential physiological relevance of transverse skeletal restriction in OSA pathophysiology [13–16]. Within this context, transverse craniofacial morphology may also influence the biomechanical response to mandibular advancement devices by modifying the anatomical relationship between the upper airway soft tissues and the surrounding craniofacial skeletal framework. Despite growing evidence supporting an association between transverse craniofacial restriction and obstructive sleep apnea, the prevalence of maxillary or maxillomandibular transverse deficiency among individuals with OSA remains unknown. This knowledge gap largely reflects the absence of standardized diagnostic criteria and the considerable heterogeneity in assessment methods across studies. Consequently, the true clinical relevance of transverse craniofacial discrepancies in OSA and their influence on treatment outcomes remain incompletely understood.

Although craniofacial morphology has long been recognized as an important determinant of obstructive sleep apnea severity and treatment response, most available evidence has focused on anteroposterior skeletal characteristics, such as maxillomandibular retrognathism. In contrast, the potential influence of transverse craniofacial discrepancies on mandibular advancement device outcomes has received considerably less attention, and the available evidence remains fragmented, heterogeneous, and difficult to interpret. A comprehensive synthesis of this literature is therefore warranted to identify current evidence, highlight knowledge gaps, and support future research aimed at improving patient selection and personalized treatment strategies for obstructive sleep apnea.

The objective of this scoping review was to systematically map and synthesize the available evidence regarding the impact of transverse craniofacial alterations on treatment response to mandibular advancement devices in adults with OSA, identify current knowledge gaps and discuss the potential clinical implications for individualized treatment selection and future research.

## Methods

### Protocol and registration

This scoping review was conducted by the Department of Otolaryngology at the University of São Paulo (São Paulo, Brazil). The protocol was developed in accordance with the methodological guidance proposed by Peters et al., for the development and reporting of scoping review protocols [17] and prospectively registered on the Open Science Framework, inscribed by https://doi.org/10.17605/OSF.IO/BJ5D6 [18]. The review was conducted following the methodological framework originally proposed by Arksey and O’Malley (2005) and subsequently refined by Peters et al. (2022) [17, 19], as outlined in the JBI Manual for Evidence Synthesis [20]. The reporting of the article complied with the PRISMA extension for Scoping Reviews (PRISMA-ScR) (Online Appendix 1) [21].

### Research question

**Fig. 1:**
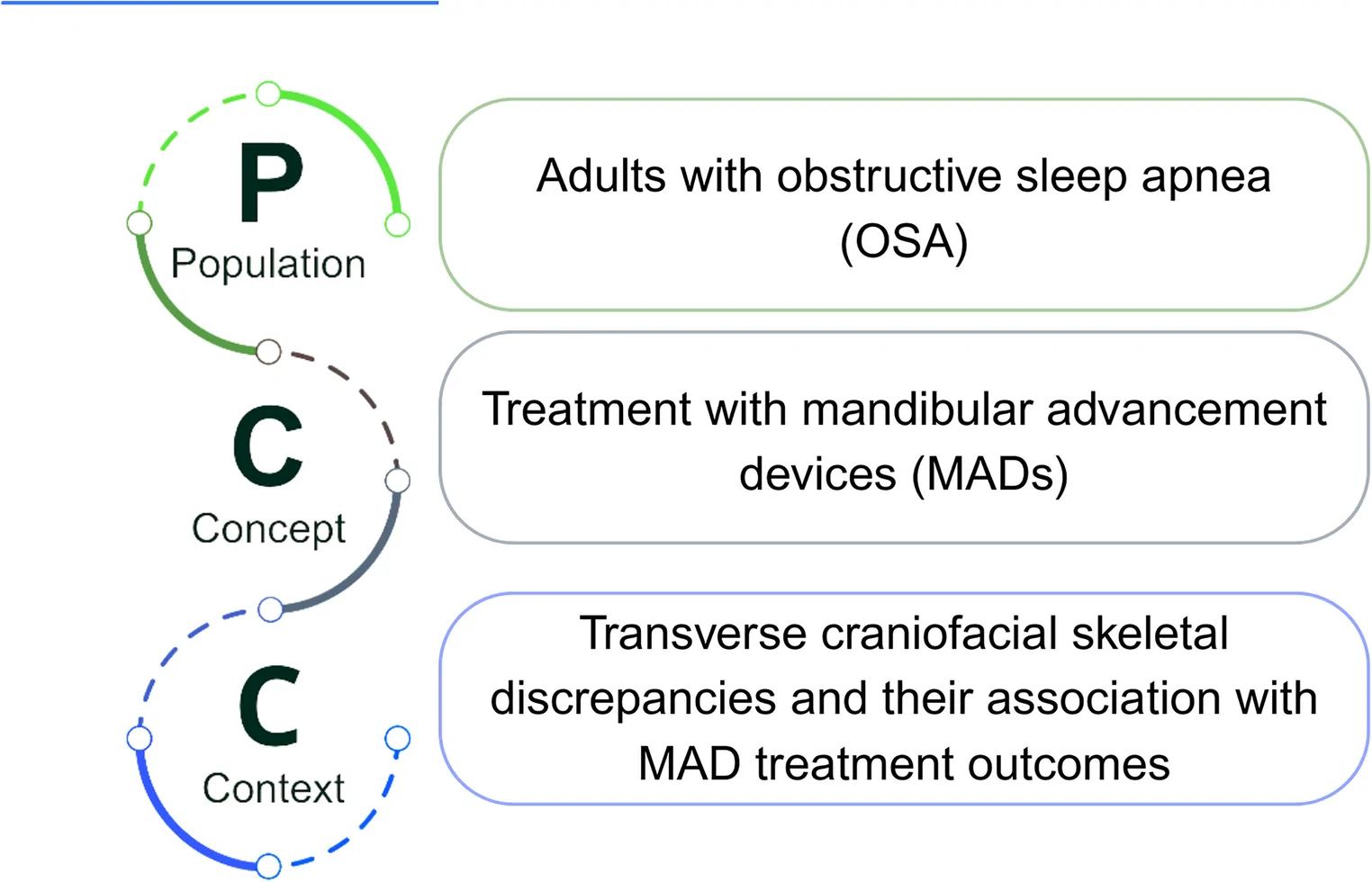
The “PCC” (Population, Concept, Context) framework used to define the eligibility criteria and guide the development of the scoping review research question. The figure was created using Canva; accessed on 16 April 2026

The research question was formulated using the PCC (Population, Concept, Context) framework (Fig. 1): “What is known about the relationship between transverse craniofacial discrepancies and the outcomes of mandibular advancement devices in adults with obstructive sleep apnea?”

### Eligibility criteria

We included studies that evaluated the impact of transverse craniofacial skeletal changes on treatment outcomes, measured as reduction in AHI, in adults over 18 years old using any mandibular advancement device. Transverse craniofacial characteristics were assessed through linear, area, volumetric, or observational measurements of the maxillae, mandible, or craniofacial skeleton. Obstructive sleep apnea (AHI > 5 events/hour) was diagnosed by type I, II, or III polysomnography and treated with mandibular advancement devices according to American Academy of Sleep Medicine criteria [2]. Clinical studies, including randomized controlled trials, non-randomized controlled trials, and before-and-after studies, as well as observational studies, such as prospective and retrospective cohort, case-control, cross-sectional, and descriptive studies, were considered. Additionally, systematic reviews that met the inclusion criteria were also considered. Studies published in any language and during any time period were eligible for inclusion. We excluded studies in which: (1) the diagnosis and evaluation of OSA (before and after MAD treatment) were performed without type I, II, or III polysomnography; (2) studies in which MAD therapy was combined with other treatment modalities.

### Search

A preliminary search strategy was developed for Medline (via PubMed) and refined in consultation with a Health Sciences Librarian. The PubMed search strategy was then adapted for use in the remaining databases (Online Appendix 2).

### Information sources

The search strategy was applied in Medline (via PubMed), Cochrane Library, Embase, LILACS (*Literatura Latinoamericana y del Caribe en Ciencias de la Salud*, via BVS), Scopus, and Web of Science Core Collection. Additionally, gray literature sources were searched through Google Scholar and ProQuest Dissertations & Theses Global. Clinical trial registries, including ClinicalTrials.gov, were also searched to identify ongoing or unpublished randomized controlled trial (RCT) protocols.

Additionally, backward and forward citation tracking was performed for all included studies using CitationChaser [22] to identify potentially relevant studies not retrieved through the initial database searches. This process involved screening the reference lists of included articles (backward citation searching) as well as studies that cited the included articles (forward citation searching). All records identified through CitationChaser were evaluated according to the predefined eligibility criteria.

Experts in the topic were also consulted by contacting the corresponding authors of the included studies via email to identify additional relevant publications, ongoing studies, or unpublished data that could contribute to the comprehensiveness of the review.

### Study selection

#### Selection process

After conducting the search, all identified references were imported and organized into the management system Endnote Online [23]. Duplicate records were removed during this process. A single file was exported to Rayyan software [24], where the selection process was carried out according to the established eligibility criteria.

In Rayyan, two authors (I.P.M.) and (B.B.D.) independently conducted a pilot test with 25 references (titles/abstracts). After that, the same authors selected the studies in two phases: titles and abstracts, and full-text, assessing their alignment with the inclusion criteria. In the first phase, titles and abstracts of all retrieved records were screened according to the predefined eligibility criteria. In the second phase, the full texts of potentially eligible studies were independently assessed to confirm their inclusion in the review. In cases of disagreement regarding the inclusion or exclusion of a study, a third reviewer (M.B.C.) was consulted, and the disagreement was resolved through discussion and consensus among the reviewers. The study selection process was detailed in the PRISMA flow diagram [25]. The excluded studies and the reasons for exclusion were organized in an Appendix entitled “Excluded Studies.”

#### Data extraction

Data extraction was carried out independently by one reviewer (I.P.M.) manually. In parallel, a second reviewer (B.B.D.) conducted the data extraction with support from an artificial intelligence (AI) tool, ChatGPT [26], to assist in the identification and organization of relevant study data [27]. Subsequently, the extracted data sets were compared and merged for the second reviewer (B.B.D.), and any discrepancies were resolved through discussion and consensus among the reviewers, and for the third reviewer (M.B.C.). When necessary, the corresponding authors of the included studies were contacted via email to obtain missing data or clarify relevant methodological information.

Before the formal data extraction process, a pilot data extraction was conducted using three evidence sources to ensure clarity, consistency, and alignment among the reviewers regarding the application of the predefined extraction criteria and the standardized data extraction form. Adjustments to the extraction process were made as necessary to improve consistency and accuracy before the full data collection phase.

The extracted data included information related to study characteristics (authors, year, country of publication, and study design), population characteristics (sample size, sex, and mean age), and the concept under investigation, including OSA severity, type of MAD, and polysomnographic criteria for MAD treatment response. Contextual data included methods used to assess transverse skeletal discrepancies, findings related to transverse skeletal discrepancies and MAD response, as well as other findings relevant to the review question. Additional information regarding study limitations, conflicts of interest, and funding sources was also collected.

## Results

### Selection of sources of evidence

**Fig. 2:**
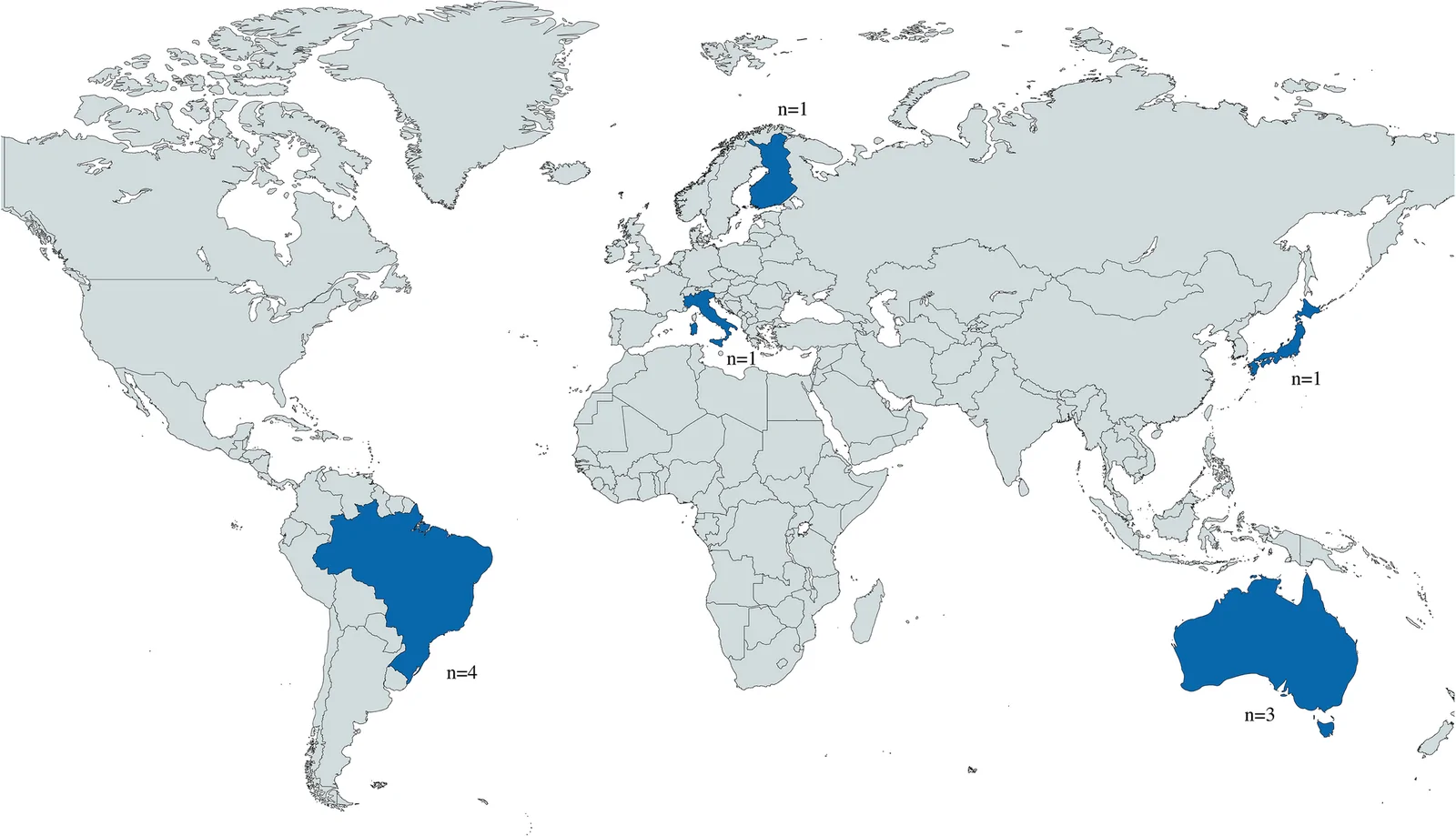
Worldwide distribution of selected studies. The figure was created using map chart; accessed on 10 April 2026

**Fig. 3:**
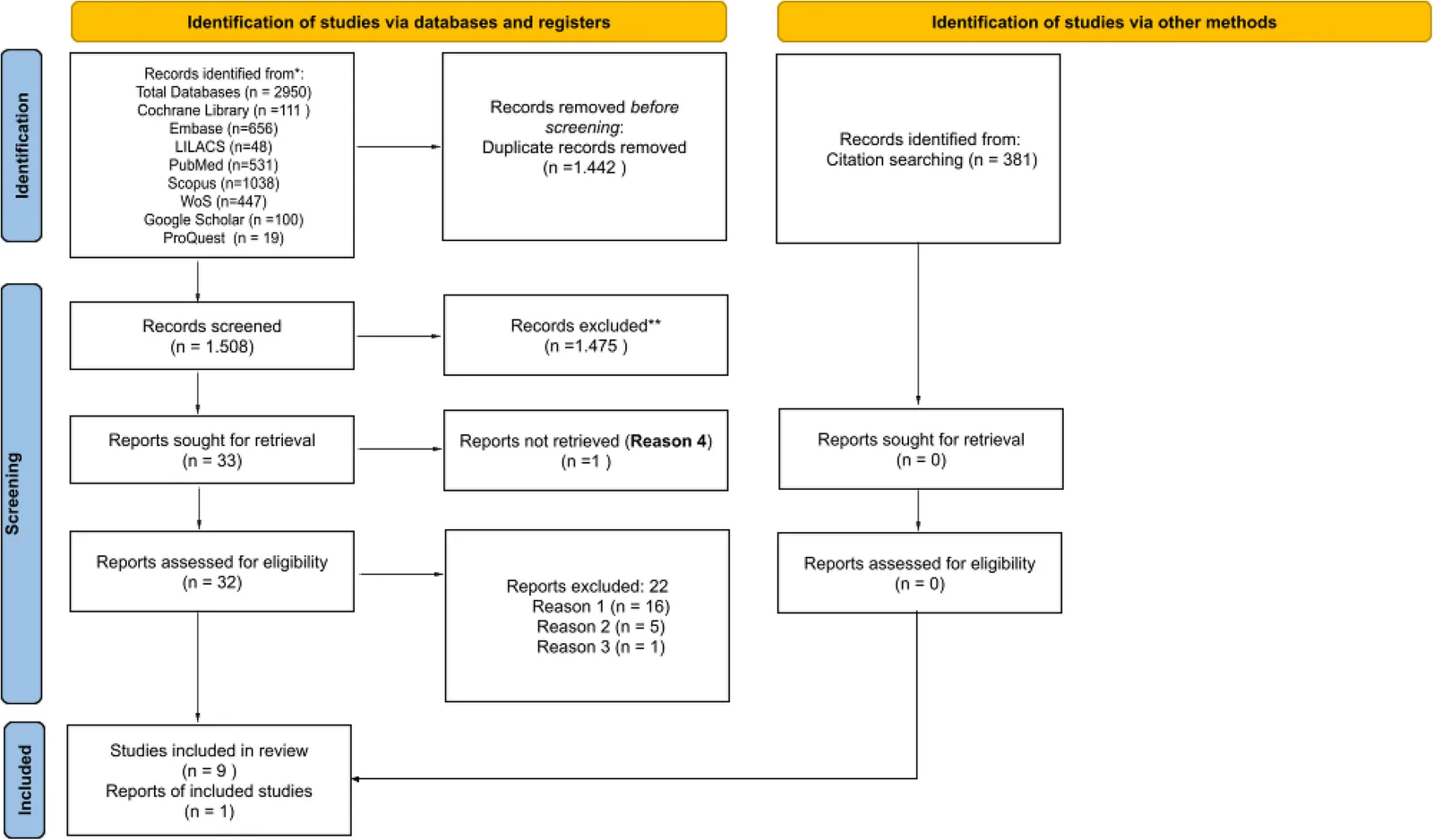
Flow diagram illustrating the study selection process, including identification, screening, eligibility assessment, and inclusion of studies, adapted according to the PRISMA guidelines

A total of 2,950 records were initially retrieved through the search strategy. After duplicate removal, 1,508 records remained for screening (phase 1). Based on title and abstract evaluation according to the predefined eligibility criteria, 32 studies were considered potentially relevant and underwent full-text assessment (phase 2). Subsequently, 22 studies were excluded after full-text review (Online Appendix 3), leading to the inclusion of nine studies and one report [28–37], whose geographic distribution is shown in Fig. 2. The snowballing strategy identified 381 additional references. Nevertheless, all studies from this supplementary search that fulfilled the eligibility criteria had already been captured in the primary search. Furthermore, consultation with experts in the field via email did not result in the identification of any further eligible studies for inclusion in this scoping review. A detailed overview of the study selection process is presented in Fig. 3.

### Characteristics of sources of evidence

The characteristics and the main findings are summarized in Table 1.

**Table 1:** Summary of characteristics of included studies (*n* = 10)

Study Characteristics		Population		Concept			Context			Other Information	
Author/Year/ Country	Study design	Sample size / male	Age (range or mean)	OSA severity (AHI range or mean, and *n* if reported)	Type of MAD	Polysomnographic criteria for MAD treatment response	Assessment of transverse skeletal discrepancy	Transverse skeletal discrepancy results and MAD response	Other findings	Limitations	Interest Conflict/ Funding
Cunha et al., 2017 — Brazil	Prospective observational study (before–after design)	40; 40	25–65 years	AHI 5–30 events/hour (12.0–18.1) / 1.5 (95%CI/SE)	Titratable custom mandibular advancement device - Brazilian Dental Appliance (BRD)	AHI < 5 events/hour and ≥ 50% AHI reduction	Dental arch model measurements (intercanine, intermolar, interpremolar widths; palate height; mouth floor depth)	Larger mandibular intercanine width was observed in responders	Better MAD response was observed in patients with milder OSA, fewer pharyngeal alterations, larger upper pharyngeal space, reduced lower airway space, and larger mandibular intercanine width; however, interdental measurements were not independent predictors of treatment success.	Small sample size with low statistical power; only non-obese males	No conflict of interest declared; support from AFIP, UNIFESP, FAPESP, CNPq
Gurgel et al., 2022 — Brazil	Prospective observational study (before–after design)	20; 9	48.35 ± 10.42 years	Mild (15), Moderate (3), Severe (2)	Titratable custom mandibular advancement device - Brazilian Dental Appliance (BRD)	AHI < 5 events/hour	CBCT craniofacial evaluation - palatal alveolar crest width (canine–molar levels), frontomaxillary/zygomaticomaxillary/frontozygomatic suture width, palatine foramen distance, nasal width, and mandibular measurements (intercondylar width, intergonial width, mandibular corpus anterior width)	No association was observed between transverse oral dimensions and MAD treatment response	Better MAD response was observed in patients with greater upper airway volume and area, whereas greater frontomaxillary transverse width was associated with lower baseline OSA severity rather than treatment response	Small sample size; absence of control group; predominance of mild OSA; limited statistical modeling; CBCT acquired in awake state	No conflict of interest declared; funding from CAPES, CNPq, NIH/NIDCR
Kohzuka et al., 2018 — Japan	Retrospective observational study (before–after design)	63; 45	57.8 years (range 35–80)	AHI 26.3 ± 14.1 events/hour	No Titratable (Monoblock) custom mandibular advancement device (~ 70% of maximum protrusion)	AHI < 5 events/hour and ≥ 50% AHI reduction	Dental arch model measurements (dental arch length - DAL, anterior dental arch width - A‑DAW, and posterior dental arch width - P‑DAW); maxilla and mandible	Larger posterior mandibular dental arch width was observed in responders	Lower baseline AHI and greater mandibular posterior dental arch width predicted OA treatment success	Retrospective design; missing dental model data for many patients	No conflicts of interest declared
Manetta et al., 2024 — Brazil	Prospective observational study (before–after design)	31; 20	Median age 49 years	AHI 18.2 (11.7; 27.6) events/h	Titratable custom mandibular advancement device	AHI < 10 events/h and > 50% AHI reduction	Dental arch model measurements (intermolar and interpremolar widths); maxilla and mandible	Smaller mandibular intermolar and interpremolar widths were observed in responders	MAD significantly reduced AHI; response associated with REMAHI < 16 events/h, favorable anatomical phenotype, low muscle responsiveness phenotype and narrower mandibular transverse width	Small sample size; indirect phenotype measurements; single-center pilot study	No conflicts of interest declared
Milano et al., 2013 — Italy	Prospective observational study (before–after design)	23; 20	54.0 ± 11.1 years	AHI 24.53 ± 13.9 events/hour	Titratable custom mandibular advancement device - Silensor^®^	AHI < 5 events/hour	Dental arch model measurements (canine, intermolar and interpremolars widths); maxilla	Smaller maxillary transverse distances associated with OSA severity, not with MAD treatment response	MAD significantly reduced AHI; maxillary transverse widths were associated with OSA severity but not MAD response; better outcomes in patients < 55 years, H-MP < 20 mm, and SN-MP < 29°.	Small sample size; cephalometry performed in upright position	No conflicts of interest declared
Mostafiz et al., 2011 — Australia	Prospective observational study (before–after design)	53; 42	49.5 ± 11.8 years	AHI 33.0 ± 14.4 events/hour	Titratable custom mandibular advancement device (SomonoDent)	AHI < 5 events/hour and > 50% AHI reduction (complete response); >50% AHI reduction (partial response)	Dental arch model measurements (intercanine, interpremolar, intermolar distances; palatal depth) maxilla and mandible	No association was observed between transverse oral dimensions and MAD treatment response	Significantly reduced AHI; no craniofacial or dental predictors identified; tongue/oral enclosure ratio (2D lateral cephalometry) associated with response	Modest sample size; tongue cross-sectional area analysis based on two-dimensional cephalometric radiographs and performed in a reduced subset of patients (*n* = 30)	Funded by National Health and Medical Research Council of Australia; SomnoMed provided appliances; some authors reported industry relationships
Mostafiz et al., 2010 — Australia	Prospective observational study (before-after design/Conference abstract)	49; not reported	not reported	Not reported (baseline AHI presented by response groups only)	Titratable custom mandibular advancement device	≥ 50% AHI reduction	Dental arch model measurements (intercanine, interpremolar, intermolar distances; palatal depth) maxilla and mandible	No association was observed between transverse oral dimensions and MAD treatment response	Age and tongue/bony enclosure ratio (2D lateral cephalometry) predicted percentage reduction in AHI	not reported	Not reported
Pahkala et al., 2020 — Finland	Prospective observational study (before–after design)	45*; 39	50.7 ± 11.9 years	AHI 19.2 ± 8.6 events/hour	Titratable custom mandibular advancement device (SomonoDent)	AHI < 5 events/hour (complete response); >50% AHI reduction (partial response)	Clinical craniofacial examination (palatal width, crossbite)	No association was observed between palatal morphology or crossbite and MAD treatment response	MAD reduced AHI and increased airway volume; younger age and deep bite favored complete response, while convex facial profile improved airway volume and increased lower facial height or vertically restricted throat were associated with poorer outcomes.	Withdrawals during follow-up; CBCT performed upright; incomplete questionnaire return; multicenter polygraphy	Funding from Finnish Dental Society Apollonia; SomnoMed provided DentiTrack system; no conflict of interest declared
Prescinotto et al., 2015 — Brazil	Prospective observational study (before–after design)	28**; 9	48.8 ± 11.3 years	AHI 17,5 ± 8,8 events/hour	Titratable custom mandibular advancement device - Brazilian Dental Appliance (BRD)	AHI ≤ 10 events/h and ≥ 50% AHI reduction	Clinical craniofacial examination (ogival palate)	No association was observed between tranverse craniofacial skeletal alterations and MAD treatment response	MAD significantly reduced AHI and ESS; younger age, smaller neck circumference, and lower baseline AHI associated with success	Small sample size; subjective ENT examination; adherence based on patient diary	No conflicts of interest declared
Sutherland et al., 2016 — Australia	Prospective observational study (before–after design)	69; 47	50.5 ± 10.1 years	AHI 27.0 ± 14.7 events/hour	Titratable custom mandibular advancement device (SomonoDent)	AHI < 10 events/h and > 50% AHI reduction	MRI craniofacial volumetric evaluation and 3D cephalometry (maxillomandibular enclosure volume; intra-mandibular area)	No association was observed between transverse maxillomandibular and intra-mandibular skeletal volumes alone and MAD treatment response	Greater soft tissue crowding relative to intra-mandibular space predicted poorer MAD response; baseline anatomical balance predicted outcome, while changes induced by MAD did not	Static awake imaging; anatomical measures may not reflect functional airway behavior or accurately assess maxillomandibular transverse constriction	Conflict of interest declared; funded by NHMRC; industry support (SomnoMed)

*58 enrolled; 45 completed follow-up PSG;

**30 enrolled; 28 completed the protocol

Abbreviations: AHI, apnea–hypopnea index; CBCT, cone-beam computed tomography; MAD, mandibular advancement device; MRI, magnetic resonance imaging; OSA, obstructive sleep apnea; CAPES, Coordenação de Aperfeiçoamento de Pessoal de Nível Superior; CNPq, Conselho Nacional de Desenvolvimento Científico e Tecnológico; NHMRC, National Health and Medical Research Council; NIH/NIDCR, National Institutes of Health/National Institute of Dental and Craniofacial Research

### Results of individual sources of evidence

In 2010 Mostafiz et al. evaluated whether craniofacial and oral cavity dimensions were associated with MAD treatment outcomes in patients with obstructive sleep apnea [33]. In this conference abstract, 49 patients treated with a customized two-piece MAD underwent cephalometric and dental cast analyses before therapy. Oral cavity dimensions obtained from dental casts did not differ between treatment responders and non-responders. However, responders showed shorter maxillary length and upper facial height, as well as a larger tongue cross-sectional area (assessed by 2D lateral cephalometry) relative to the bony oral enclosure. Multiple regression analysis identified age and the tongue-to-bony enclosure ratio as significant predictors of AHI reduction with MAD therapy. The authors suggested that cephalometric assessment of anatomical balance between tongue volume and oral enclosure size may help predict response to oral appliance treatment.

Building upon these preliminary findings, the same authors subsequently conducted a prospective study [34] involving 53 patients with OSA treated with a customized two-piece MAD to further investigate the relationship between craniofacial anatomy, oral cavity dimensions, and treatment response. Cephalometric analyses and dental cast measurements were performed to evaluate whether craniofacial structure, oral cavity dimensions, and anatomical balance were associated with treatment outcomes. Consistent with the previous conference abstract, intertooth distances, palatal dimensions, and oral cavity measurements did not significantly differ between responders and non-responders. However, complete responders presented a larger tongue area (2D lateral cephalometric tongue cross-sectional area relative to the bony oral enclosure), as well as shorter maxillary length and upper facial height compared with partial responders. Larger tongue area was associated with greater AHI reduction, and multiple regression analysis identified younger age and tongue area as variables associated with treatment outcome. The findings suggest that the relationship between tongue size and bony oral enclosure may contribute to MAD treatment response.

In 2013, Milano et al. evaluated whether anthropometric, occlusal, and craniofacial characteristics were associated with MAD treatment efficacy in 23 adults with mild to severe OSA treated with a Silensor^®^ appliance. Dental cast analyses included assessment of sagittal and transverse occlusal relationships, overjet, overbite, and maxillary intercanine, interpremolar, and intermolar distances, while cephalometric evaluation assessed skeletal and airway-related variables. The transverse maxillary dimensions demonstrated fair-to-good negative correlations with baseline AHI, such that reduced maxillary widths were associated with greater OSA severity. However, transverse dental measurements did not differ significantly between responders and non-responders after MAD therapy. Better MAD response was observed in younger patients and in those with smaller H-MP distances and lower mandibular plane divergence (SN^MP). The results indicate that reduced maxillary transverse dimensions may contribute to OSA severity, whereas specific craniofacial characteristics may be associated with better response to MAD treatment [32].

Two years later, Prescinotto et al. (2015) evaluated whether upper airway and craniofacial abnormalities were associated with MAD treatment success and adherence in 28 adults with mild to moderate OSA treated with a titratable customized MAD. Upper airway and craniofacial assessments included nasal, pharyngeal, and facial skeletal alterations identified through systematic clinical examination. MAD treatment success was observed in 64.3% of patients, whereas good adherence was identified in 60.7%. Nasal abnormalities were more frequently observed in patients with treatment failure, while pharyngeal and craniofacial skeletal abnormalities were not significantly associated with treatment success or adherence. Better MAD response was observed in younger patients and in those with smaller neck circumference and lower baseline AHI. This may indicate that nasal obstruction may negatively influence MAD treatment efficacy, whereas craniofacial skeletal alterations identified by clinical examination were not independently associated with treatment outcomes [36].

Subsequently, Sutherland et al. (2016) evaluated whether three-dimensional anatomical balance between upper airway soft tissues and maxillomandibular skeletal enclosure was associated with MAD treatment outcomes in 69 adults with OSA treated with a customized two-piece MAD. Magnetic resonance imaging (MRI) and three-dimensional cephalometric analyses were performed to assess upper airway soft tissue volumes, maxillomandibular enclosure volume, and intra-mandibular area. Soft tissue volumes did not significantly differ between responders and non-responders. However, non-responders presented higher soft tissue-to-intra-mandibular area ratios, indicating greater anatomical imbalance within the mandibular enclosure. Higher soft tissue-to-intra-mandibular area ratios were independently associated with poorer MAD treatment response. In contrast, soft tissue-to-maxillomandibular volume ratios and changes in anatomical balance produced by mandibular advancement were not associated with MAD treatment outcomes. The authors suggested that excess upper airway soft tissue relative to intra-mandibular space may negatively influence MAD treatment response, whereas static imaging measures alone may not fully reflect functional upper airway improvement [37].

In 2017, Cunha et al. evaluated whether demographic, anthropometric, polysomnographic, otorhinolaryngological, cephalometric, and dental model variables were associated with MAD treatment success in 40 non-obese men with mild to moderate OSA treated with a titratable customized MAD. Cephalometric analyses included assessment of upper and lower pharyngeal airway spaces, while dental model evaluation included intercanine, interpremolar, and intermolar widths, maxillary and mandibular lengths, palate height, and mouth floor depth. Responders presented fewer pharyngeal alterations, larger upper pharyngeal space, smaller lower airway space, and greater mandibular intercanine width compared with non-responders. Better MAD response was also observed in patients with milder OSA. However, logistic and linear regression analyses did not identify independent predictors of treatment success. The results support that greater upper airway patency and larger mandibular transverse dimensions were associated with better MAD treatment response, although isolated anatomical and functional variables were insufficient to reliably predict treatment outcomes [28].

Kohzuka et al. (2018) evaluated whether dental arch dimensions were associated with MAD treatment response in 63 patients with OSA treated with a monobloc oral appliance. Dental cast analyses included upper and lower dental arch length, anterior dental arch width, and posterior dental arch width measurements. Responders were defined as patients presenting both a ≥ 50% reduction in AHI and a post-treatment AHI < 5 events/hour. Lower posterior dental arch width was significantly greater in responders compared with non-responders, whereas other dental arch measurements did not significantly differ between groups. Lower baseline AHI and greater lower posterior dental arch width were independently associated with better treatment response in multivariate logistic regression analysis. The authors suggested that wider mandibular transverse dimensions may reflect greater pharyngeal airway width and contribute to improved OA treatment outcomes [30].

In 2020, Pahkala et al. evaluated whether upper airway volume changes and craniofacial characteristics were associated with MAD treatment outcomes in 58 adults with OSA treated with a customized titratable MAD with moderate mandibular advancement. Clinical assessment included occlusal and craniofacial evaluation, while cone-beam computed tomography (CBTC) was used to assess upper airway volume and minimal cross-sectional airway areas with and without the MAD in situ. Complete responders were younger and presented deeper overbite compared with partial/non-complete responders. Greater upper airway volume increase was observed in patients with a convex facial profile, whereas increased lower facial height and a vertically restricted throat were negatively associated with airway volume increase. MAD therapy significantly increased total upper airway volume and minimal cross-sectional airway areas, particularly in the oropharyngeal region. However, craniofacial and upper airway variables were not independently associated with treatment response in logistic regression analysis. The results suggest that craniofacial morphology may influence upper airway dimensional changes during MAD therapy, although isolated anatomical characteristics were insufficient to reliably predict treatment outcomes [35].

After these, Gurgel et al. (2022) evaluated whether three-dimensional craniofacial and upper airway characteristics were associated with OSA severity and MAD treatment outcomes in 20 adults with OSA treated with a titratable customized MAD. CBCT was used to assess maxillary, mandibular, and upper airway linear, angular, area, and volumetric measurements before and after MAD therapy. Greater transverse width at the frontomaxillary suture was associated with lower baseline AHI, whereas greater mandibular ramus facial angle was associated with higher OSA severity. MAD therapy significantly reduced AHI and increased superior oropharyngeal volume and area. Upper airway total area was associated with AHI improvement, while greater upper airway volume and area measurements were associated with greater therapeutic protrusion required during MAD therapy. However, most maxillary and mandibular transverse, linear, angular, and volumetric measurements were not independently associated with treatment response. The authors suggested that specific craniofacial transverse and upper airway characteristics may influence OSA severity and the amount of mandibular advancement required during MAD therapy, although isolated anatomical variables were insufficient to reliably predict treatment outcomes [29].

Finally, in 2024 Manetta et al. evaluated whether pathophysiological OSA phenotypes, morpho-anthropometric characteristics, and polysomnographic variables were associated with MAD treatment response in 31 adults with OSA treated with a customized two-piece MAD. Morpho-anthropometric assessment included cephalometric analyses and transverse dental arch measurements, such as maxillary and mandibular intermolar and interpremolar distances. Polysomnographic phenotypes related to anatomical traits, ventilatory control, arousal threshold, and muscle responsiveness were estimated from baseline PSG data. Better MAD response was observed in patients with lower REM-related apnea-hypopnea index, more favorable anatomical phenotype scores, and worse muscle responsiveness phenotype scores. Smaller mandibular intermolar and interpremolar distances were also associated with better treatment response, suggesting a relationship between mandibular transverse constriction and MAD treatment outcomes. However, cephalometric variables, mandibular advancement amount, and most anthropometric characteristics were not significantly associated with treatment response. These findings suggest that polysomnographic phenotyping combined with morpho-anthropometric evaluation may improve prediction of MAD treatment outcomes, although isolated anatomical variables were insufficient to reliably predict treatment success [31].

### Synthesis of results

**Fig. 4:**
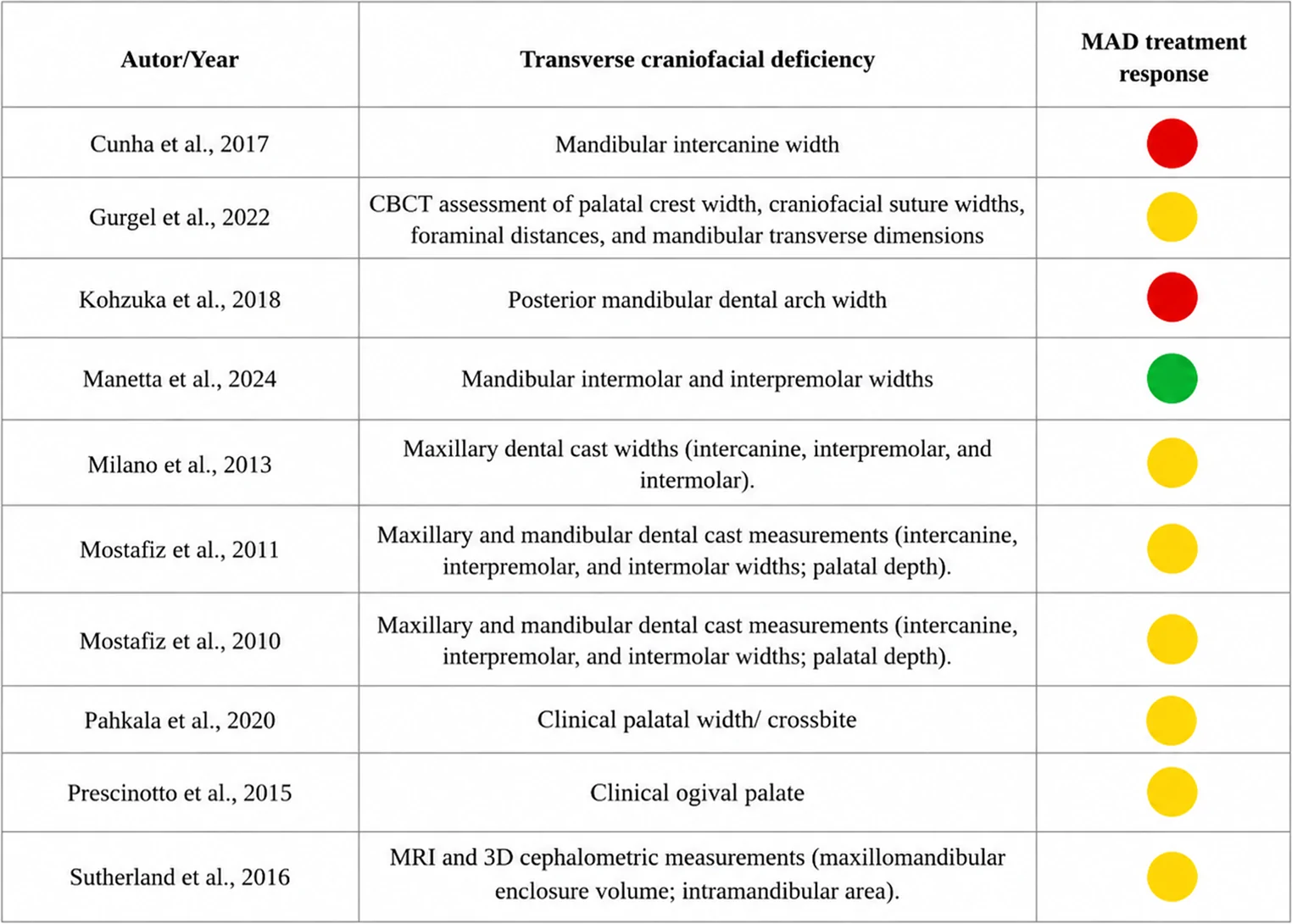
Summary of transverse craniofacial deficiency parameters assessed across included studies and their corresponding response to MAD treatment. The color-coded circles represent treatment response classification (red: unfavorable; yellow: neutral or inconclusive; green: favorable). The figure was created using *Keynote*

The available evidence demonstrated heterogeneous associations between transverse craniofacial characteristics and MAD treatment outcomes. Within the sample included in this scoping review, six studies and one conference report (70%) did not identify significant associations between the evaluated transverse craniofacial variables and response to MAD therapy. Greater mandibular transverse dimensions were associated with better treatment response in two studies (20%), suggesting that increased mandibular width may favor upper airway dimensions and contribute to improved therapeutic outcomes. In contrast, one study (10%) reported an inverse association, observing better MAD treatment response in individuals with smaller mandibular transverse dimensions. Reduced maxillary width and unfavorable anatomical balance were more frequently associated with greater OSA severity or poorer therapeutic outcomes. Overall, most studies did not identify independent associations between isolated transverse craniofacial measurements and MAD treatment success, suggesting that treatment response is multifactorial and influenced by both anatomical and non-anatomical factors. A synthesis of the transverse craniofacial findings and their relationship with MAD treatment response is presented in Fig. 4.

## Discussion

In this scoping review, we performed an extensive literature search to identify the best available evidence on the association between transverse craniofacial skeletal alterations and treatment outcomes of MAD in adults with OSA. Despite a broad search strategy involving electronic databases, grey literature, clinical trial registries, AI-assisted tools, and citation and reference screening, the available evidence regarding the relationship between transverse craniofacial discrepancies and MAD treatment response was limited to nine observational studies and one conference report.

Craniofacial morphology plays a crucial role in both OSA severity and the effectiveness of MAD therapy [38]. This review reinforces that, although several studies have demonstrated associations between anteroposterior craniofacial characteristics, such as maxillomandibular retrognathism, and both OSA severity and MAD treatment response [39, 40], these relationships remain poorly investigated in the context of transverse skeletal alterations.

Although several anteroposterior cephalometric features—such as shorter maxillary length, reduced anterior and posterior facial height, decreased hyoid-to-C3 distance, shorter airway length, and smaller minimum airway cross-sectional area—have been associated with better MAD treatment response [39, 40], two-dimensional cephalometric measurements remain relatively weak and inconsistent predictors, limiting their isolated use in clinical decision-making [41].

More recently, the understanding of MAD treatment response has progressively shifted from isolated craniofacial measurements toward broader anatomical and pathophysiological phenotyping approaches. Three-dimensional craniofacial analyses and individualized response models suggest that treatment efficacy is likely determined by the interaction between multiple anatomical traits rather than by single linear measurements alone [15, 42, 43]. In this context, craniofacial morphology should be interpreted as part of a multidimensional framework that includes upper airway anatomy, soft tissue balance, obesity, ventilatory control stability, arousal threshold, and upper airway muscle responsiveness. Therefore, transverse craniofacial dimensions may represent only one component within a more complex anatomical phenotype associated with MAD responsiveness.

Regarding transverse craniofacial characteristics, previous studies have demonstrated an association between maxillary transverse deficiency and OSA severity [14, 32, 44]. In this context, Thuler et al. (2023) further reinforced the concept that transverse skeletal restriction is associated with increased pharyngeal collapsibility during sleep, supporting a potential role of transverse craniofacial deficiency in the pathogenesis of upper airway obstruction [13]. Nevertheless, evidence investigating the relationship between transverse craniofacial alterations and response to non-surgical therapies, such as MAD treatment, remains scarce.

The selected studies presented heterogeneous findings. Most studies did not identify significant associations between transverse craniofacial characteristics and response to MAD treatment [29, 32–37]. However, two studies (Cunha et al., 2017; Kohzuka et al., 2018) reported better treatment response in individuals with less mandibular transverse deficiency (atresia), suggesting that greater mandibular transverse dimensions may favor increased pharyngeal airway width and contribute to improved oral appliance treatment outcomes. Only one study [31] identified an inverse relationship between smaller distances between the lower posterior teeth (mandibular atresia) and better response to MAD treatment, possibly due to greater anatomical pharyngeal obstruction associated with reduced lower airway space caused by lingual retropositioning, a factor known to improve with MAD therapy [45].

The search for clinically applicable predictors to identify better responders to MAD therapy is useful and relevant, considering that approximately one-third of patients do not achieve complete response to this treatment [46] and could therefore avoid prolonged periods without adequate disease control as well as the financial burden associated with therapy. The marked heterogeneity among studies on this topic has limited the establishment of consistent findings, and these variables are still rarely incorporated into broader phenotypic analyses. Improved characterization of transverse craniofacial features may contribute to the development of more individualized therapeutic approaches targeting specific anatomical traits.

Contemporary predictive models increasingly support the concept of individualized therapy in obstructive sleep apnea, integrating craniofacial morphology with clinical, polysomnographic, and pathophysiological phenotypes [31, 47]. In this scenario, isolated transverse craniofacial measurements may have limited predictive value when evaluated independently, whereas combined anatomical and functional phenotyping approaches may better identify patients more likely to benefit from MAD therapy. Future studies should therefore prioritize multidimensional predictive models capable of integrating craniofacial structure, upper airway collapsibility, ventilatory control traits, and sleep-related physiological characteristics.

Furthermore, anatomical assessments should be interpreted within a broader clinical context that includes factors such as age, sex, and Body Mass Index (BMI) [41], as well as combined non-anatomical physiological factors and associated comorbidities [48]. This broader perspective is also consistent with evidence emphasizing the relevance of lifestyle-related factors and cardiometabolic comorbidities in patients with OSA, supporting the integration of anatomical assessment within a more comprehensive clinical phenotype [49].

From a clinical perspective, the findings of this review suggest that transverse craniofacial assessment should not be considered an isolated predictor of mandibular advancement device treatment success. Instead, transverse skeletal characteristics should be interpreted alongside established anatomical, polysomnographic, and pathophysiological phenotypes when selecting candidates for oral appliance therapy. Although current evidence does not support the routine use of isolated transverse craniofacial measurements for predicting treatment outcomes, these features may contribute to a more comprehensive individualized assessment and support precision medicine approaches in obstructive sleep apnea management.

This review also presents several methodological strengths. To our knowledge, it is the first scoping review specifically designed to synthesize the evidence regarding the influence of transverse craniofacial discrepancies on mandibular advancement device treatment outcomes in obstructive sleep apnea. The review protocol was prospectively registered, conducted according to the Joanna Briggs Institute methodological guidance, and reported following the PRISMA extension for Scoping Reviews. In addition, the comprehensive search strategy, including multiple electronic databases, gray literature, clinical trial registries, citation tracking, and consultation with experts, minimizes the likelihood of missing relevant evidence and provides a robust framework for future research in this field. An additional limitation relates to the inherent nature of the available evidence. Most included studies were observational and therefore do not allow causal inferences regarding the relationship between transverse craniofacial discrepancies and mandibular advancement device treatment outcomes. Furthermore, the substantial heterogeneity in study design, imaging techniques, craniofacial measurements, and definitions of treatment success precluded quantitative synthesis, limiting the interpretation of the available evidence to a narrative approach.

Future research should prioritize well-designed prospective multicenter studies using standardized definitions of transverse craniofacial deficiency and harmonized protocols for craniofacial assessment. Integrating three-dimensional imaging, upper airway functional assessment, polysomnographic phenotyping, and other pathophysiological traits may improve the identification of patients most likely to benefit from mandibular advancement device therapy. Such multidimensional approaches may contribute to the development of more accurate predictive models and support individualized treatment selection in obstructive sleep apnea.

## Limitations

The evidence included in this scoping review consisted predominantly of prospective observational studies with small sample sizes and heterogeneous methodologies, lacking the robustness required for definitive clinical validation. A notable absence of standardized clinical protocols for the assessment of transverse craniofacial deformities was observed, limiting comparability across studies. Assessment methods varied considerably, ranging from objective measures, such as dental arch dimensions and CBCT/MRI analyses, to more subjective clinical findings, including high-arched palate and posterior crossbite. These anatomical domains are not equivalent and may reflect distinct biological and functional phenomena. Consequently, comparisons among studies should be interpreted with caution, as transverse dental dimensions do not necessarily correspond to the functional behavior of the upper airway. In addition, baseline obstructive sleep apnea (OSA) severity and the cutoff criteria used to define therapeutic success with mandibular advancement devices (MADs) were inconsistent across studies.

Collectively, these findings suggest that isolated transverse craniofacial measurements may have limited predictive value, reinforcing the notion that response to MAD therapy is likely multifactorial and influenced by a complex interaction of anatomical and non-anatomical factors. Therefore, future research should prioritize the development of standardized craniofacial assessment protocols, as well as prospective studies with larger sample sizes and more homogeneous methodologies, in order to improve the clinical applicability and predictive utility of these markers in identifying patients who are more likely to respond to MAD treatment.

## Conclusions

Current evidence regarding the impact of transverse craniofacial alterations on MAD treatment outcomes in adults with OSA remains scarce, heterogeneous, and predominantly based on observational studies with small sample sizes. Although some studies suggested associations between mandibular transverse dimensions and treatment response, most investigations did not identify independent relationships between isolated transverse craniofacial measurements and MAD treatment success, reinforcing that treatment response is likely multifactorial and influenced by a complex interaction between anatomical and non-anatomical factors. Nevertheless, understanding how transverse craniofacial alterations correlate with MAD treatment outcomes may contribute to a more comprehensive phenotypic characterization of patients with OSA and support individualized therapeutic decision-making. Rather than acting as isolated predictors, transverse craniofacial alterations may represent part of broader anatomical and pathophysiological phenotypes associated with upper airway collapsibility and MAD responsiveness. Future research should therefore move beyond isolated linear measurements and prioritize integrated multidimensional phenotyping models capable of combining craniofacial morphology, upper airway anatomy, and non-anatomical physiological traits.

## Supplementary Information

Below is the link to the electronic supplementary material.

Supplementary Material 1 (Docx 72.2 KB)

Supplementary Material 2 (Docx 12.7 KB)

Supplementary Material 3 (Docx 54.2 KB)

## Data Availability

Data will be made available on reasonable request.

## Abbreviations

AHI: apnea–hypopnea index
AI: artificial intelligence
BMI: body mass index
CAPES: Coordenação de Aperfeiçoamento de Pessoal de Nível Superior
CBCT: cone-beam computed tomography
ClinicalTrials.gov: ClinicalTrials.gov registry
CNPq: Conselho Nacional de Desenvolvimento Científico e Tecnológico
CPAP: continuous positive airway pressure
MAD: mandibular advancement device
MRI: magnetic resonance imaging
OSA: obstructive sleep apnea
PCC: Population, Concept, Context
PRISMA: Preferred Reporting Items for Systematic Reviews and Meta-Analyses
PRISMA-ScR: Preferred Reporting Items for Systematic Reviews and Meta-Analyses extension for Scoping Reviews
PSG: polysomnography
RCT: randomized controlled trial
