# Supplementary material for: Impact of maxillomandibular atresia on the response to mandibular advancement devices in obstructive sleep apnea: a scoping review
Source: Sleep Breath. 2026 Sep 25;30(5):271. doi: 10.1007/s11325-026-03783-z (PMC13615085; doi:10.1007/s11325-026-03783-z)
Supplement: Supplementary file 2 [file 11325_2026_3783_MOESM2_ESM.docx]

**Appendix 1** - Databases and search strategies.

| Database | Search  strategy | Results  2950 August 12^th^ 2025 |
| --- | --- | --- |
| Medline / PubMed | ("intercanine distance"[Title/Abstract:~5] OR "intercanine distances"[Title/Abstract:~5] OR "intermolar distance"[Title/Abstract:~5] OR "interpremolar distance"[Title/Abstract:~5] OR "intermolar distances"[Title/Abstract:~5] OR "interpremolar distances"[Title/Abstract:~5] OR "intercanine width"[Title/Abstract:~5] OR "intermolar width"[Title/Abstract:~5] OR "interpremolar width"[Title/Abstract:~5] OR "interdental distance"[Title/Abstract:~5] OR "interdental width"[Title/Abstract:~5] OR "intercanine widths"[Title/Abstract:~5] OR "intermolar widths"[Title/Abstract:~5] OR "interpremolar widths"[Title/Abstract:~5] OR "interdental distances"[Title/Abstract:~5] OR "interdental widths"[Title/Abstract:~5] OR "skeletal facial"[Title/Abstract:~5] OR "cranial facial"[Title/Abstract:~5] OR "craniofacial"[Title/Abstract] OR "atresia"[Title/Abstract] OR "transversal"[Title/Abstract] OR "transverse"[Title/Abstract] OR "occlusal"[Title/Abstract] OR "Dental Arch"[MeSH Terms] OR "dental arch*"[Title/Abstract] OR "malocclusion*"[Title/Abstract] OR "crossbite*"[Title/Abstract] OR "cross bite*"[Title/Abstract] OR "Cephalometry"[MeSH Terms] OR "cephalometr*"[Title/Abstract] OR "craniometr*"[Title/Abstract]) AND ("Occlusal Splints"[MeSH Terms] OR "occlusal splint*"[Title/Abstract] OR "mandibular advancing device*"[Title/Abstract] OR "MAD"[Title/Abstract] OR "MAA"[Title/Abstract] OR "MADs"[Title/Abstract] OR "MAMA"[Title/Abstract] OR "oral appliance"[Title/Abstract] OR "OA"[Title/Abstract] OR "Mandibular Advancement"[MeSH Terms] OR "Mandibular Advancement"[Title/Abstract] OR "dental appliance"[Title/Abstract]) AND ("sleep apnea, obstructive"[MeSH Terms] OR "obstructive sleep apnea*"[Title/Abstract] OR "obstructive sleep apnoea"[Title/Abstract] OR "OSAHS"[Title/Abstract] OR "Sleep Apnea Hypopnea"[Title/Abstract:~3] OR "Upper Airway Resistance Sleep Apnea"[Title/Abstract:~3] OR "OSAS"[Title/Abstract] OR "OSA"[Title/Abstract] OR "SDB"[Title/Abstract] OR "Sleep Apnea Syndromes"[MeSH Terms:noexp] OR "sleep hypopnea*"[Title/Abstract] OR "Hypersomnia with Periodic Respiration"[Title/Abstract] OR "Sleep Disordered Breathing"[Title/Abstract]) | 531 |
| Embase | ((('intercanine' NEAR/6 'distance'):ti,ab,kw) OR (('intercanine' NEAR/6 'distances'):ti,ab,kw) OR (('intermolar' NEAR/6 'distance'):ti,ab,kw) OR (('interpremolar' NEAR/6 'distance'):ti,ab,kw) OR (('intermolar' NEAR/6 'distances'):ti,ab,kw) OR (('interpremolar' NEAR/6 'distances'):ti,ab,kw) OR (('intercanine' NEAR/6 'width'):ti,ab,kw) OR (('intermolar' NEAR/6 'width'):ti,ab,kw) OR (('interpremolar' NEAR/6 'width'):ti,ab,kw) OR (('interdental' NEAR/6 'distance'):ti,ab,kw) OR (('interdental' NEAR/6 'width'):ti,ab,kw) OR (('intercanine' NEAR/6 'widths'):ti,ab,kw) OR (('intermolar' NEAR/6 'widths'):ti,ab,kw) OR (('interpremolar' NEAR/6 'widths'):ti,ab,kw) OR (('interdental' NEAR/6 'distances'):ti,ab,kw) OR (('interdental' NEAR/6 'widths'):ti,ab,kw) OR (('skeletal' NEAR/6 'facial'):ti,ab,kw) OR (('cranial' NEAR/6 'facial'):ti,ab,kw) OR 'craniofacial':ti,ab,kw OR 'atresia':ti,ab,kw OR 'transversal':ti,ab,kw OR 'transverse':ti,ab,kw OR 'occlusal':ti,ab,kw OR 'tooth arch'/exp OR 'dental arch*':ti,ab,kw OR 'malocclusion*':ti,ab,kw OR 'crossbite*':ti,ab,kw OR 'cross bite*':ti,ab,kw OR 'cephalometry'/exp OR 'cephalometr*':ti,ab,kw OR 'craniometr*':ti,ab,kw) AND ('occlusal splint'/exp OR 'occlusal splint*':ti,ab,kw OR 'mandibular advancing device*':ti,ab,kw OR 'mad':ti,ab,kw OR 'maa':ti,ab,kw OR 'mads':ti,ab,kw OR 'mama':ti,ab,kw OR 'oral appliance':ti,ab,kw OR 'oa':ti,ab,kw OR 'mandibular advancement'/exp OR 'mandibular advancement':ti,ab,kw OR 'dental appliance':ti,ab,kw) AND ('obstructive sleep apnea'/exp OR 'obstructive sleep apnea*':ti,ab,kw OR 'obstructive sleep apnoea':ti,ab,kw OR 'osahs':ti,ab,kw OR (('sleep' NEAR/4 'apnea' NEAR/4 'hypopnea'):ti,ab,kw) OR (('upper' NEAR/4 'airway' NEAR/4 'resistance' NEAR/4 'sleep' NEAR/4 'apnea'):ti,ab,kw) OR 'osas':ti,ab,kw OR 'osa':ti,ab,kw OR 'sdb':ti,ab,kw OR 'sleep apnea syndromes'/de OR 'sleep hypopnea*':ti,ab,kw OR 'hypersomnia with periodic respiration':ti,ab,kw OR 'sleep disordered breathing':ti,ab,kw) | 656 |
| Scopus | TITLE-ABS-KEY((intercanine W/5 distance*) OR (intermolar W/5 distance*) OR (interpremolar W/5 distance*) OR (intercanine W/5 width*) OR (intermolar W/5 width*) OR (interpremolar W/5 width*) OR (interdental W/5 distance*) OR (interdental W/5 width*) OR (skeletal W/5 facial) OR (cranial W/5 facial) OR craniofacial OR atresia OR transversal OR transverse OR occlusal OR "dental arch*" OR Malocclusion* OR Crossbite* OR "Cross Bite*" OR Cephalometr* OR Craniometr*) AND TITLE-ABS-KEY("Occlusal Splint*" OR "mandibular advancing device*" OR "MAD" OR "MAA" OR "MADs" OR "MAMA" OR "oral appliance" OR "OA" OR "Mandibular Advancement" OR "dental appliance") AND TITLE-ABS-KEY("Obstructive Sleep Apnea*" OR "obstructive sleep apnoea" OR "OSAHS" OR ("Sleep Apnea" W/3 Hypopnea) OR ("Upper Airway Resistance" W/3 "Sleep Apnea") OR "OSAS" OR "OSA" OR "SDB" OR "Sleep Apnea Syndromes" OR "Sleep Hypopnea*" OR "Hypersomnia with Periodic Respiration" OR "Sleep Disordered Breathing") | 1,038 |
| Web of Science | TS=((intercanine NEAR/5 distance*) OR (intermolar NEAR/5 distance*) OR (interpremolar NEAR/5 distance*) OR (intercanine NEAR/5 width*) OR (intermolar NEAR/5 width*) OR (interpremolar NEAR/5 width*) OR (interdental NEAR/5 distance*) OR (interdental NEAR/5 width*) OR (skeletal NEAR/5 facial) OR (cranial NEAR/5 facial) OR craniofacial OR atresia OR transversal OR transverse OR occlusal OR "dental arch*" OR Malocclusion* OR Crossbite* OR "Cross Bite*" OR Cephalometr* OR Craniometr*) AND TS=("Occlusal Splint*" OR "mandibular advancing device*" OR "MAD" OR "MAA" OR "MADs" OR "MAMA" OR "oral appliance" OR "OA" OR "Mandibular Advancement" OR "dental appliance") AND TS=("Obstructive Sleep Apnea*" OR "obstructive sleep apnoea" OR "OSAHS" OR ("Sleep Apnea" NEAR/3 Hypopnea) OR ("Upper Airway Resistance" NEAR/3 "Sleep Apnea") OR "OSAS" OR "OSA" OR "SDB" OR "Sleep Apnea Syndromes" OR "Sleep Hypopnea*" OR "Hypersomnia with Periodic Respiration" OR "Sleep Disordered Breathing") | 447 |
| Cochrane Library | ((intercanine NEAR/5 distance*):ti,ab,kw OR (intermolar NEAR/5 distance*):ti,ab,kw OR (interpremolar NEAR/5 distance*):ti,ab,kw OR (intercanine NEAR/5 width*):ti,ab,kw OR (intermolar NEAR/5 width*):ti,ab,kw OR (interpremolar NEAR/5 width*):ti,ab,kw OR (interdental NEAR/5 distance*):ti,ab,kw OR (interdental NEAR/5 width*):ti,ab,kw OR (skeletal NEAR/5 facial):ti,ab,kw OR (cranial NEAR/5 facial):ti,ab,kw OR craniofacial:ti,ab,kw OR atresia:ti,ab,kw OR transversal:ti,ab,kw OR transverse:ti,ab,kw OR occlusal:ti,ab,kw OR [mh "Dental Arch"] OR (dental NEXT arch*):ti,ab,kw OR Malocclusion*:ti,ab,kw OR Crossbite*:ti,ab,kw OR (Cross NEXT Bite*):ti,ab,kw OR [mh "Cephalometry"] OR Cephalometr*:ti,ab,kw OR Craniometr*:ti,ab,kw) AND ([mh "Occlusal Splints"] OR (Occlusal NEXT Splint*):ti,ab,kw OR (mandibular NEXT advancing NEXT device*):ti,ab,kw OR "MAD":ti,ab,kw OR "MAA":ti,ab,kw OR "MADs":ti,ab,kw OR "MAMA":ti,ab,kw OR "oral appliance":ti,ab,kw OR "OA":ti,ab,kw OR [mh "Mandibular Advancement"] OR "Mandibular Advancement":ti,ab,kw OR "dental appliance":ti,ab,kw) AND ([mh "sleep apnea, obstructive"] OR ("Obstructive Sleep Apnea"):ti,ab,kw OR "obstructive sleep apnoea":ti,ab,kw OR "OSAHS":ti,ab,kw OR "Sleep Apnea Hypopnea":ti,ab,kw OR "Upper Airway Resistance Sleep Apnea":ti,ab,kw OR "OSAS":ti,ab,kw OR "OSA":ti,ab,kw OR "SDB":ti,ab,kw OR [mh "Sleep Apnea Syndromes"] OR "Sleep Apnea Syndromes":ti,ab,kw OR (Sleep NEXT Hypopnea*):ti,ab,kw OR "Hypersomnia with Periodic Respiration":ti,ab,kw OR "Sleep Disordered Breathing":ti,ab,kw) | 111 |
| LILACS | ("intercanine distance" OR "intercanine distances" OR "intermolar distance" OR "interpremolar distance" OR "intermolar distances" OR "interpremolar distances" OR "intercanine width" OR "intermolar width" OR "interpremolar width" OR "interdental distance" OR "interdental width" OR "intercanine widths" OR "intermolar widths" OR "interpremolar widths" OR "interdental distances" OR "interdental widths" OR "skeletal facial" OR "cranial facial" OR craniofacial OR atresia OR transversal OR transverse OR occlusal OR "Dental Arch" OR "dental arches" OR malocclusion OR malocclusions OR crossbite OR crossbites OR "cross bite" OR "cross bites" OR cephalometry OR craniometry OR "Arco Dental" OR "Arcada Dental" OR "Arcada Dentária" OR "Arco Dentário" OR cefalometria OR craniometria OR "Circunferência Craniana" OR "Perímetro Cefálico" OR craneometría) AND ("Occlusal Splints" OR "occlusal splint" OR "mandibular advancing device" OR "mandibular advancing devices" OR "MAD" OR "MAA" OR "MADs" OR "MAMA" OR "oral appliance" OR "OA" OR "Mandibular Advancement" OR "dental appliance" OR "Placas Oclusais" OR "Placas Miorrelaxantes" OR "Placas de Mordida" OR "Ferulas Oclusales" OR "férula dental" OR "Avanço Mandibular" OR "Avance Mandibular" OR "adelantamiento mandibular" OR "Avanzamiento Mandibular" ) AND ("obstructive sleep apnea" OR "obstructive sleep apnoea" OR "OSAHS" OR "Sleep Apnea Hypopnea" OR "Upper Airway Resistance Sleep Apnea" OR "OSAS" OR "OSA" OR "SDB" OR "Sleep Apnea" OR "sleep hypopnea" OR "Hypersomnia with Periodic Respiration" OR "Sleep Disordered Breathing" OR "Apneia Obstrutiva do Sono" OR "Apneia do Sono" OR "Apnea Obstructiva del Sueño" OR "apnea del sueño") AND db:("LILACS") AND instance:"regional" | 48 |
| ProQuest Dissertations & Theses Citation Index | TS=((intercanine NEAR/5 distance*) OR (intermolar NEAR/5 distance*) OR (interpremolar NEAR/5 distance*) OR (intercanine NEAR/5 width*) OR (intermolar NEAR/5 width*) OR (interpremolar NEAR/5 width*) OR (interdental NEAR/5 distance*) OR (interdental NEAR/5 width*) OR (skeletal NEAR/5 facial) OR (cranial NEAR/5 facial) OR craniofacial OR atresia OR transversal OR transverse OR occlusal OR "dental arch*" OR Malocclusion* OR Crossbite* OR "Cross Bite*" OR Cephalometr* OR Craniometr*) AND TS=("Occlusal Splint*" OR "mandibular advancing device*" OR "MAD" OR "MAA" OR "MADs" OR "MAMA" OR "oral appliance" OR "OA" OR "Mandibular Advancement" OR "dental appliance") AND TS=("Obstructive Sleep Apnea*" OR "obstructive sleep apnoea" OR "OSAHS" OR ("Sleep Apnea" NEAR/3 Hypopnea) OR ("Upper Airway Resistance" NEAR/3 "Sleep Apnea") OR "OSAS" OR "OSA" OR "SDB" OR "Sleep Apnea Syndromes" OR "Sleep Hypopnea*" OR "Hypersomnia with Periodic Respiration" OR "Sleep Disordered Breathing") | 19 |
| Google Scholar | ("intercanine distance" OR "intermolar distance" OR "interpremolar distance" OR "interdental distance" OR "intercanine width" OR "intermolar width" OR "interdental width" OR craniofacial OR transversal OR "Dental Arch" OR malocclusion OR crossbite OR cephalometry OR "Arcada Dentária") AND ("occlusal splint" OR "mandibular advancing device" OR "oral appliance" OR "Mandibular Advancement" OR "dental appliance" OR "Placas Oclusais" OR "Avanço Mandibular" OR "férula dental") AND ("obstructive sleep apnea" OR "OSA" OR "Sleep Apnea Hypopnea" OR "Sleep Disordered Breathing" OR "Apneia Obstrutiva do Sono" OR "apnea del sueño") | 100 |

Search strategies were performed for each database by using specifics words combinations and truncations with the support of a librarian.
