# Supplementary material for: Impact of maxillomandibular atresia on the response to mandibular advancement devices in obstructive sleep apnea: a scoping review
Source: Sleep Breath. 2026 Sep 25;30(5):271. doi: 10.1007/s11325-026-03783-z (PMC13615085; doi:10.1007/s11325-026-03783-z)
Supplement: Supplementary file 3 [file 11325_2026_3783_MOESM3_ESM.docx]

**Additional file 3**

Appendix 3 Excluded articles and reasons for exclusion.

| **Author,**  **Year** | **Reason for exclusion** |
| --- | --- |
| Abe, S et al 2019^1^ | 1 |
| Balasubramaniam, R et al 2024^2^ | 1 |
| Bamagoos, A et al 2015^3^ | 1 |
| Camañes-Gonzalvo, S et al 2025^4^ | 1 |
| Camañes-Gonzalvo, S et al 2025^5^ | 1 |
| Cebola, P et al 2023^6^ | 1 |
| Cebola P et al 2025^7^ | 1 |
| Chen H et al 2019^8^ | 1 |
| Haskell BS et al 2021^9^ | 1 |
| Heiser C 2019^10^ | 1 |
| Kalha, A et al 2024^11^ | 2 |
| Lee, R. W. et al 2010^12^ | 2 |
| Lowe, A. A. 1993^13^ | 1 |
| Ma, Y et al 2023^14^ | 1 |
| Mayoral Sanz, P et al 2015^15^ | 1 |
| Navarro, R. E et al 2024^16^ | 1 |
| Ogawa, T 2022^17^ | 1 |
| Ogawa, T et al 2015^18^ | 1 |
| Pintucci, F et al 2024^19^ | 2 |
| Scarfe, W 2009^20^ | 3 |
| Teixeira, A. O. B et al 2018 ^21^ | 2 |
| Uniken Venema, J. A. M et al 2023^22^ | 2 |

1- No assessment of transverse craniofacial skeletal alterations (n=16), 2- No analysis of the association between transverse craniofacial skeletal alterations and MAD treatment response (n=5), 3- No post-treatment polysomnography to acess MAD response (n=1), 4- Clinical trialdiscontinued or still ongoing (no published resulats available) (n=1)

**References**

1.	Abe, S., Mori, H., Tanaka, E., Tachibana, N., Murakami, A., Okura, K., Suzuki, Y., Okawa, T., Kawano, F. Craniofacial morphology in OSA patients treated by oral appliance with and without sufficient effects. *Sleep Medicine* - Volume 64, Issue 0, pp. S1-S2 - published 2019-12-01

2.	Balasubramaniam, R., McCloy, K., Almeida, F. and Cistulli, P. Oral appliance therapy for snoring and obstructive sleep apnoea: a Practical Guide for Clinical Care. *Aust Dent J*. 2024, 69: S84-S100.

3.	Bamagoos, A., Sutherland, K., Chan, A., Leigh, M., Ngiam, J., Darendeliler, M. A., Cistulli, P. Photographic craniofacial analysis and mandibular advancement splint treatment response, Abstracts. *Sleep and Biological Rhythms*. 2015, 13: 1-98.

4.	Camañes-Gonzalvo, S., Montiel-Company, J.M., García-Selva, M., Plaza-Espín, A., Pérez-Carbonell, T., Paredes-Gallardo, V., Bellot-Arcís, C. and Marco-Pitarch, R. Individualised Therapy for Obstructive Sleep Apnoea: Predictive Models and Anatomical Phenotyping of Mandibular Advancement Devices Responses. *Orthod Craniofac Res*. 2025, 28: 485-495.

5.	Camañes-Gonzalvo, S., Montiel-Company, J.M., Marco-Pitarch, R., Plaza-Espín, A., Paredes-Gallardo, V., Bellot-Arcís, C. and García-Selva, M. Association of Skeletal Facial Pattern With Treatment Response of Obstructive Sleep Apnoea Using Mandibular Advancement Devices – A Cluster Analysis. *J Oral Rehabil*, 2025, 52: 957-968.

6.	Cebola, P.; Caroça, C.; Donato, H.; Campos, A.; Dias, S.S.; Paço, J.; Manso, C. Computed Tomography versus Sleep Endoscopy (DISE) to Predict the Effectiveness of Mandibular Advancement Devices in Adult Patients with Obstructive Sleep Apnea: A Protocol for Systematic Review. J. Clin. Med. 2023, 12, 6328.

7.	Cebola P, Caroça C, Donato H, Dias S, Paço J, Manso C. Computed tomography versus sleep endoscopy (DISE) to predict the effectiveness of mandibular advancement device in adult patients with obstructive sleep apnea: A systematic review. *PLoS One*. 2025 Jul 10;20(7): e0327974.

8.	Chen H, Aarab G, Lobbezoo F, De Lange J, Van der Stelt P, Darendeliler MA, Cistulli PA, Sutherland K, Dalci O. Differences in three-dimensional craniofacial anatomy between responders and non-responders to mandibular advancement splint treatment in obstructive sleep apnoea patients. *Eur J Orthod.* 2019 May 24;41(3):308-315.

9.	Haskell BS, Voor MJ, Roberts AM. A consideration of factors affecting palliative oral appliance effectiveness for obstructive sleep apnea: a scoping review. *J Clin Sleep Med.* 2021 Apr 1;17(4):833-848.

10.	Heiser C. Phenotypes to Predict Response to Mandibular Advancement Device Therapy. *J Clin Sleep Med.* 2019 Aug 15;15(8):1073-1074.

11.	Kalha, A. S., Raju, S. Airway-centric orthodontics: a review on oral appliance therapy as a simplified solution to obstructive sleep apnea. *Frontiers of Oral and Maxillofacial Medicine*. 2024, Volume 6, Issue 0.

12.	Richard W. W. Lee, Kate Sutherland, Andrew S. L. Chan, Biao Zeng, Ronald R. Grunstein, M. Ali Darendeliler, Richard J. Schwab, Peter A. Cistulli, Relationship Between Surface Facial Dimensions and Upper Airway Structures in Obstructive Sleep Apnea, Sleep, Volume 33, Issue 9, September 2010, Pages 1249–1254.

13.	Alan A. Lowe, Can We Predict the Success of Dental Appliance Therapy for the Treatment of Obstructive Sleep Apnea Based on Anatomic Considerations. *Sleep*, Volume 16, Issue suppl_8, December 1993, Pages S93–S95.

14.	Ma Y, Yu M, Gao X. Role of craniofacial phenotypes in the response to oral appliance therapy for obstructive sleep apnea. *J Oral Rehabil.* 2023; 50:308-317.

15.	Mayoral Sanz, P., Calvo, E. Three-dimensional cone-beam computed tomography for assessment of airway changes with mandibular advance splint. *Sleep Medicine* - Volume 14, Issue 0, pp. e257 - published 2013-01-01.

16.	Navarro, R. E., Karadede, B., Karadede Ünal, B., Salvador, D. M. Predictive factors of therapeutic response according to craniofacial skeletal biotype in patients with sleep apnea syndrome using mandibular advancement devices: a pilot study. *Angle Orthod -* Volume 94, Issue 2, pp. 216-223 - published 2024-03-01

17.	Toru Ogawa, Prediction of mandibular advancement splint treatment response: moving toward individualized therapy for obstructive sleep apnea, *Sleep*, Volume 45, Issue 6, June 2022, zsac072.

18.	Ogawa, T., Long, J., Sutherland, K. *et al.* Effect of mandibular advancement splint treatment on tongue shape in obstructive sleep apnea. *Sleep Breath*. 2015; 19, 857–863.

19.	Pintucci F, Cremonini F, Romagnolo G, Marrano GG, Barbanti F, Spedicato GA, Vicini C, Lombardo L. Effectiveness of Mandibular Advancement Devices in Positional OSA Patients: A Retrospective Analysis of Predictive Variables in a Sample of Adult Patients. *Sleep Sci.* 2024 Feb 20;17(1): e55-e63.

20.	Scarfe, W. Assessment of the effects of Mandibular Advancement Device (MAD) on airway dimensions using cone-beam computed tomography. *Sleep and Biological Rhythms* - Volume 7, Issue 0, pp. A3 - published 2009-01-01.

21.	Teixeira AOB, Andrade ALL, Almeida RCDC, Almeida MAO. Side effects of intraoral devices for OSAS treatment. *Braz J Otorhinolaryngol.* 2018 Nov-Dec;84(6):772-780. doi: 10.1016/j.bjorl.2017.09.003. Epub 2017 Oct 14. PMID: 29102398; PMCID: PMC9442828.

22.	Uniken Venema JAM, Bosschieter PFN, Hoekema A, Plooij JM, Lobbezoo F, de Vries N. Do dental parameters predict severity of obstructive sleep apnea and mandibular advancement device therapy outcomes? A pilot study. *J Oral Rehabil.* 2023;50:203-209.
